# StructuralVariantAnnotation: a R/Bioconductor foundation for a caller-agnostic structural variant software ecosystem

**DOI:** 10.1093/bioinformatics/btac042

**Published:** 2022-02-04

**Authors:** Daniel L Cameron, Ruining Dong, Anthony T Papenfuss

**Affiliations:** The Walter and Eliza Hall Institute of Medical Research, Parkville, VIC 3052, Australia; Department of Medical Biology, University of Melbourne, Parkville, VIC 3052, Australia; The Walter and Eliza Hall Institute of Medical Research, Parkville, VIC 3052, Australia; Department of Medical Biology, University of Melbourne, Parkville, VIC 3052, Australia; The Walter and Eliza Hall Institute of Medical Research, Parkville, VIC 3052, Australia; Department of Medical Biology, University of Melbourne, Parkville, VIC 3052, Australia; Bioinformatics and Cancer Genomics Laboratory, Peter MacCallum Cancer Centre, East Melbourne, VIC 3002, Australia; Sir Peter MacCallum Department of Oncology, University of Melbourne, Melbourne, VIC 3010, Australia

## Abstract

**Summary:**

StructuralVariantAnnotation is an R/Bioconductor package that provides a framework for decoupling downstream analysis of structural variant breakpoints from upstream variant calling methods. It standardizes the representational format from BEDPE, or any of the three different notations supported by VCF into a *breakpoint GRanges* data structure suitable for use by the wider Bioconductor ecosystem. It handles both transitive breakpoints and duplication/insertion notational differences of identical variants—both common scenarios when comparing short/long read-based call sets that confound downstream analysis. StructuralVariantAnnotation provides the caller-agnostic foundation needed for a R/Bioconductor ecosystem of structural variant annotation, classification and interpretation tools able to handle both simple and complex genomic rearrangements.

**Availability and implementation:**

StructuralVariantAnnotation is implemented in R and available for download as the Bioconductor StructuralVariantAnnotation package. Details can be found at https://www.bioconductor.org/packages/release/bioc/html/StructuralVariantAnnotation.html. It has been released under a GPL license.

## 1 Breakpoint-centric design philosophy

**Fig. 1.**
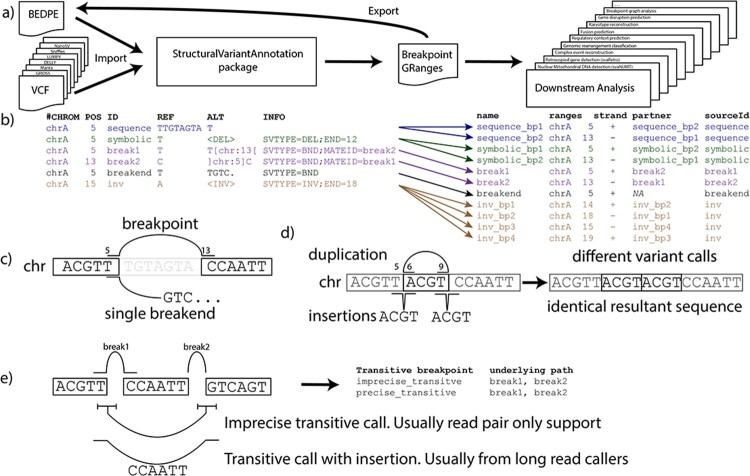
StructuralVariantAnnotation provides a potential foundation for a caller-agnostic structural variant tool ecosystem. (**a**) StructuralVariantAnnotation simplifies downstream analysis by decoupling the caller from the analysis. (**b**) Structural variants are converted to a common breakpoint GRanges notation regardless of how they are represented in VCF. Strand indicates breakend orientation. Inversions are decomposed into their constituent breakpoints. (**c**) Breakpoints are composed of a pair of connected breakends. Single breakend variants are breakpoints where only one side can be placed. (**d**) Long read callers frequently report duplications as insertions. StructuralVariantAnnotation can identify when an insertion and duplication represent the same genomic rearrangement. (**e**) StructuralVariantAnnotation can identify transitive breakpoints—spurious breakpoints that span two or more breakpoints in a complex genomic rearrangement

While simple rearrangements such as insertions, deletions and duplications can, for many purposes, be analyzed using techniques and tools similar or identical to those used for indels, restricting structural variant analysis to just these events belie the heterogeneity and complexity that can occur in genomic rearrangements. Simple events such as deletions can in fact form part of more complex events such as chromothripsis or retrocopied transcripts. To fully understand the nature and impact of genomic rearrangements, we need an entire ecosystem of analysis, classification, interpretation and annotation tools (Fig. 1a). While sophisticated analysis tools do exist (Hadi *et al.*, 2020; Shale *et al.*, 2020), they are tightly coupled to their specific variant callers. StructuralVariantAnnotation provides a potential foundation for the development of a R/Bioconductor ecosystem of such tools that could be applied to the output of most general-purpose SV callers.

Fundamentally, genomic rearrangements are identified from the combination of copy number segments and breakpoints. While copy number segments are easily and naturally represented using the GenomicRanges Bioconductor package (Lawrence *et al.*, 2013), representing breakpoints is less straight-forward. Although classes like InteractionSet (Lun *et al.*, 2016) and Pairs (Pagès *et al.*, 2017) represent pairs of genomic coordinates, they are not designed for representing structural variants in a manner that supports ease of integration with existing Bioconductor annotation and analysis packages, the ability to compare equivalent structural variants regardless of the VCF notation the variant is represented in, nor do they support single breakend variants (Cameron *et al.*, 2021). StructuralVariantAnnotation takes the approach of decomposing all structural variant calls into their constituent breakpoints. This has a number of advantages: it enables standardized analysis across callers that use different VCF notations when reporting variants; it simplifies the rearrangement data model to its most fundamental form; supports arbitrarily complex rearrangements; is fully compatible with the upcoming structural variant changes in version 4.4 of VCF; it ensures both sides of each breakpoint are treated identically; it simplifies the annotation of breakpoints and it provides a foundation for sophisticated downstream processes such as breakpoint graph analysis.

## 2 Implementation

### 2.1 Breakpoint GRanges data format

StructuralVariantAnnotation uses a lightweight paired GRanges notation in which each breakend is a row in a GRanges object. Mirroring the VCF (Danecek *et al.*, 2011) BND/breakpoint notation, breakpoints are represented as pairs of genomics coordinates, with the other side of the breakpoint identified by a column (MATEID for VCF, partner for StructuralVariantAnnotation) containing the unique name of the other side. Single breakends are supported through the use of NA partner values, and multibreakpoint variant calls such as inversions are decomposed into their constituent breakpoints (Fig. 1c). This notation uses approximately 512 bytes per breakpoint—<30% of the memory of the corresponding VariantAnnotation::VCF object.

Since breakends are stored in a GRanges object, operations such as annotating in which gene or repeat a breakend lies can be done using the standard Bioconductor annotation packages.

#### 2.1.1 Uncertainty around breakend location

Breakend positions can be ambiguous for one of two reasons. First, the variant call itself may be imprecise and the position unambiguous but not known precisely. Second, homologous sequences at the two breakend results in multiple breakpoints positions having identical resultant sequences. In such scenarios, the breakpoint is known precisely but there remains uncertainty around the nominal breakpoint position.

StructuralVariantAnnotation encodes both forms of uncertainty by specifying each breakend position not as a single position, but as the interval over which the breakend could be located. The interval used is that reported by the variant caller. For BEDPE the interval is taken directly from the start1/start2 and end1/end2 columns and for VCF is inferred from ALT sequence and CIPOS, CILEN, CIEND, HOMSEQ, HOMLEN and HOMPOSINFO fields. A HOMLEN greater than 0 in the breakpoint GRanges object indicates a precise call with homology.

### 2.2 Support for all VCF notations

The VCF specifications (Danecek *et al.*, 2011) allow structural variants to be represented in many different notations. Popular callers make use of different notations for identical variants. Simple variants can be represented in the direct sequence notation or through one of the four specifications-defined structural variant symbolic alleles that can represent breakpoints: <DEL>, <DUP>, <INS> and <INV>. Intrachromosomal variants can and interchromosomal variants must be represented in BND/breakpoint notation. Finally, VCF has yet another notation for single breakend variants.

StructuralVariantAnnotation is built on top of the VariantAnnotation package (Obenchain *et al.*, 2014) and introduces the breakpointRanges() and breakendRanges() functions to convert from the various VCF notations into breakpoint GRanges notations (Fig. 1b). Note that <DEL> and <DUP> are treated as breakpoint (not copy number) variant calls and inversions are decomposed into their constituent breakpoints.

StructuralVariantAnnotation supports loading variants from VCF, BEDPE and S4Vectors::Pairs objects. It includes logic for parsing TRA, RPL, CTX, CHR2, INV3/INV5, UNK, IMPRECISE_DIR variants from DELLY, TIGRA, Pindel, Manta, LongRanger and other callers that are not compliant with the VCF specifications. At the time of writing, StructuralVariantAnnotation has been tested and works with BreakDancer (via the VCF conversion script), CLEVER, Cortex, CREST, DELLY, Dindel, GRIDSS, GRIDSS2, Hydra, LongRanger, LUMPY, Manta, NanoSV, NovoBreak, PBSV, Pindel, Socrates, Sniffles, SvABA, TIGRA and Weaver.

### 2.3 Matching variants

Mirroring the GenomicRanges findOverlaps() function, StructuralVariantAnnotation provides an equivalent findBreakpointOverlaps() function, which identifies which breakpoints are equivalent (based on a configurable error margin). Basic comparison between call sets is as simple as:

>truth = breakpointRanges(readVcf(“truth.vcf”))

>caller = breakpointRanges(readVcf(“caller.vcf”))

>caller$tp = countBreakpointOverlaps(truth, caller) > 0

By default, an exact positional match is required (uncertain positions are considered to be an exact match to any position in the interval) but more lenient matching criteria can be specified using optional parameters. Breakpoints are considered to be matching if the position and orientation of the two breakends in each breakpoint match. This differs from tools such as SVanalyzer, Truvari (Zook *et al.*, 2020) or Parliament (English *et al.*, 2015) which all first classify by type (e.g. deletion/duplication/inversion) then compare bounds and length within type. This approach gives StructuralVariantAnnotation the unique ability to match all breakpoints independent of their representation in VCF but does mean that relative and absolute event size matching criteria only apply to intrachromosomal breakpoints such as deletions and duplications.

StructuralVariantAnnotation is a library and not itself a fully fledged analysis/benchmarking tool. Capabilities such as rearrangement classification, ensemble calling, benchmarking (Cameron *et al.*, 2019) and sequencing-based variant matching logic (Cameron *et al.*, 2021) can be implemented on top of the StructuralVariantAnnotation library.

#### 2.3.1 Matching duplications and insertions

Even after standardizing variant calls in a breakpoint GRanges notation, some variants will be incorrectly reported as mismatching. Most notable is the failure to match moderately sized (50–1000 bp) duplications with their equivalent insertion syntax. Duplications around this size range are typically reported as insertions by long read callers and duplications by short read callers (Fig. 1d). Directly comparing short and long read call sets will result in a higher mismatch rate than is actually the case. StructuralVariantAnnotation provides the findInsDupOverlaps() function to identify these equivalent variants reported in different notations.

#### 2.3.2 Matching transitive variants

Another source of spurious mismatching between call sets are transitive breakpoints. Transitive breakpoints are rearrangements that are reported as a single breakpoint but are in fact composed of multiple breakpoints (Fig. 1e). There are two sources of transitive breakpoints: imprecise breakpoints and precise breakpoints with sequence inserted at the breakpoint. The former is typically called by read-pair based short read variant callers, the latter by long read-based callers. Transitive calls occur when two or more breakpoints occur in close proximity on the derivative chromosome—typically less than a few hundred base pairs between breakpoints.

The presence of transitive breakpoints has two impacts: spurious mismatching between short and long read call sets, and the confounding of derivative chromosome reconstruction. StructuralVariantAnnotation provides findTransitiveCalls() to identify transitive calls and report the underlying set of breakpoints corresponding to the transitive call.

## 3 Conclusion

StructuralVariantAnnotation provides a unified breakpoint GRanges notation for the representation of breakpoints in the R/Bioconductor ecosystem. StructuralVariantAnnotation supports all specifications compliant VCF representation formats as well as the non-standard notations used by many popular structural variant callers. It allows easy comparison of call sets from callers using different VCF notations. Uniquely, it can match equivalent variants reported as insertion and duplication and can identify transitive breakpoints. Such features are important as they are common when comparing short and long read call sets.

StructuralVariantAnnotation provides a potential foundation for a variant caller-agnostic ecosystem of analysis, classification, interpretation and annotation tools in R/Bioconductor. Methods using StructuralVariantAnnotation that identify retrocopied transcripts and nuclear mitochondrial insertions [svaRetro and svaNUMT (Dong *et al.*, 2021), respectively] that are already available in Bioconductor.

## Funding

A.T.P. was supported by a National Health and Medical Research Council (NHMRC) Senior Research Fellowship [1116955] and the Lorenzo and Pamela Galli Charitable Trust. D.L.C. and A.T.P. was supported by an NHMRC Ideas Grant [1188098]. The research benefited by support from the Victorian State Government Operational Infrastructure Support and Australian Government NHMRC Independent Research Institute Infrastructure Support.

*Conflict of Interest*: none declared.

## References

[btac042-B1] Cameron D.L. et al (2019) Comprehensive evaluation and characterisation of short read general-purpose structural variant calling software. Nat. Commun., 10, 3240. 31324872 10.1038/s41467-019-11146-4 PMC6642177

[btac042-B2] Cameron D.L. et al (2021) GRIDSS2: comprehensive characterisation of somatic structural variation using single breakend variants and structural variant phasing. Genome Biol., 22, 202. 34253237 10.1186/s13059-021-02423-x PMC8274009

[btac042-B3] Danecek P. et al (2011) The variant call format and VCFtools. Bioinformatics, 27, 2156–2158. 21653522 10.1093/bioinformatics/btr330 PMC3137218

[btac042-B4] Dong R. et al (2021) svaRetro and svaNUMT: modular packages for annotation of retrotransposed transcripts and nuclear integration of mitochondrial DNA in genome sequencing data. *bioRxiv*. https://bioconductor.org/packages/release/bioc/html/svaNUMT.html. 10.46471/gigabyte.70 PMC9694029 36824522

[btac042-B5] English A.C. et al (2015) Assessing structural variation in a personal genome—towards a human reference diploid genome. BMC Genomics, 16, 286. 25886820 10.1186/s12864-015-1479-3 PMC4490614

[btac042-B6] Hadi K. et al (2020) Distinct classes of complex structural variation uncovered across thousands of cancer genome graphs. Cell, 183, 197–210.e32. 33007263 10.1016/j.cell.2020.08.006 PMC7912537

[btac042-B7] Lawrence M. et al (2013) Software for computing and annotating genomic ranges. PLoS Comput. Biol., 9, e1003118. 23950696 10.1371/journal.pcbi.1003118 PMC3738458

[btac042-B8] Lun A.T.L. et al (2016) Infrastructure for genomic interactions: Bioconductor classes for Hi-C, ChIA-PET and related experiments. F1000Research, 5, 950. 27303634 10.12688/f1000research.8759.1 PMC4890298

[btac042-B9] Obenchain V. et al (2014) VariantAnnotation: a Bioconductor package for exploration and annotation of genetic variants. Bioinformatics, 30, 2076–2078. 24681907 10.1093/bioinformatics/btu168 PMC4080743

[btac042-B10] Pagès H. et al (2017) S4Vectors: S4 implementation of vectors and lists, R package version 0.13, https://bioconductor.org/packages/release/bioc/html/S4Vectors.html.

[btac042-B11] Shale C. et al (2021) Unscrambling cancer genomes via integrated analysis of structural variation and copy number. *Cell Genomics*10.1016/j.xgen.2022.100112 PMC9903802 36776527

[btac042-B12] Zook J.M. et al (2020) A robust benchmark for detection of germline large deletions and insertions. Nat. Biotechnol., 38, 1347–1355. 32541955 10.1038/s41587-020-0538-8 PMC8454654

